# The lack of specialized pediatric cardiac surgeons in Lebanon: a humanitarian catastrophe

**DOI:** 10.1186/s13561-023-00424-z

**Published:** 2023-02-11

**Authors:** Jana Zeineddine, Carolla El Chamieh, Elie Bou Sanayeh

**Affiliations:** 1 Faculty of Medicine, American University of Beirut, Beirut, Lebanon; 2 Faculty of Medicine, Sorbonne University, Paris, France; 3 Faculty of Medicine, Department of Internal Medicine, American University of Beirut, Beirut, Lebanon

**Keywords:** Lebanese healthcare system, Pediatric cardiac surgeries, Public health crisis

## Abstract

Congenital heart disease (CHD) is a major public health concern, as it is the most common birth defect and the leading cause of death in the first year of life if adequate surgical interventions were not provided. Unfortunately, in Lebanon, a country that has been assailed by devastating social and economic crises, many specialized Lebanese pediatric heart surgeons fled abroad to secure more stable careers. This has led to the death of many newborns with CHDs. Public health authorities must find urgent solutions for this national tragedy that is projected to last for years.

## Background

Despite its long history of corruption and socioeconomic instability, Lebanon has always upheld a highly respected healthcare system. Housing some of the most brilliant medical minds, Beirut has attracted international patients for the past several decades. However, nothing could have prepared Lebanon for its recent downfall. According to the World Bank, Lebanon’s economic crisis falls under the top three crises globally, since the nineteenth century, with an inflation rate of over 131.9% over the first 6 months of 2021 [1]. From social unrest to the devaluation of the Lebanese pound, every facet of life has shifted. This goes without mentioning the massive Port Explosion on the 4^th^ of August 2020, which left citizens questioning the feasibility of life in Lebanon [2]. Importantly, this soon became reflected in Lebanon’s medical system. A prime example lies within the specialty of pediatric cardiology [3].

## Pediatric cardiac surgery

Four pediatric cardiac surgery centers exist in Lebanon, including the American University of Beirut Medical Center, Hotel Dieu Hospital, Hammoud Hospital and Beirut Heart Center [3]. However, over the last three years the overwhelming majority of pediatric heart surgeons fled to secure more stable careers in the United States of America (USA) and Europe, leaving Lebanon with only eight pediatric cardiologists and five pediatric cardiac surgeons [4]. This led to the passing of four pediatric patients over a timespan of two weeks in February of 2022 [3]. To combat this undeniable issue, specialized surgeons have volunteered to return on a weekly or monthly basis to perform necessary cardiac surgeries. Similarly, many Non-governmental organizations (NGO) have come together in support of financially-disabled patients, offering funds to cover costly medical treatments. However, this has proven insufficient for many reasons. To begin with, this fails to account for emergency cases which simply cannot wait for the arrival of competent surgeons. Moreover, Lebanon’s economic crisis has forced most patients under the poverty line and the available NGO funds are not sufficient to cover medical bills in their entirety, which average at $27,450 ± $13,685 per procedure [4]. This fact is exacerbated by the maldistribution of available funds. Therefore, many patients remain unable to afford treatment regardless of surgeon availability and financial assistance. Thus, families are left with no choice but to delay life-saving surgeries, producing even more unnecessary deaths. Finally, Lebanese hospitals are no longer able to secure the appropriately sized pediatric medical supplies and equipment, making it even more difficult to perform successful surgeries.

Meanwhile, Congenital Heart Disease (CHD) is a major public health concern as it is the most common birth defect and the leading cause of death in the first year of life. One in every 100 babies is born with congenital heart defects [5]. Consequently, approximately 600 Lebanese babies are diagnosed with heart disease every year [5]. Furthermore, 400 of these require either surgical or non-surgical interventions to enable their survival. Without proper care, 70% of these patients will not make it to their first birthday, while 1/3 will die before 1 month of life [5]. In sharp contrast, with adequate treatment, 95% of these babies can grow to lead normal lives [5]. Though CHD is prevalent across the globe, the burden associated seems to be concentrated in countries with unfavorable socioeconomic conditions and insufficient surgical workforces. However, the impact of the socioeconomic collapse on CHD treatment in Lebanon is especially significant due to the rapid, unexpected deterioration that left little time for preparation or adaptation.

## Further limitations

On top of the existing shortage in pediatric cardiac surgeons, patients are limited by further obstacles. First, the economic decline in Lebanon produced shortages in a variety of medications, which is detrimental for both surgical and non-surgical candidates [1]. In addition, with the 90% drop of the local currency’s value, healthcare workers’ salaries have taken a pitfall [2]. This left the majority of nurses, residents, physicians and surgeons poorly compensated for their efforts, resulting in significant drops in motivation levels among healthcare providers [2]. Unfortunately, this further deteriorates the quality of care offered to patients, in general. Further, hospitals commonly face a shortage of resuscitative equipment commonly used during cardiothoracic surgeries such as airway and suction devices or other intraoperative monitoring systems [4]. Additionally, even well-established centers have unreliable electricity, water and oxygen supplies, all of which impede sound medical care.

## Moving forwards

The threats faced by our medical systems are only expected to amplify. A cross-sectional, multicentered study was conducted to assess physicians’, clinical fellows’, residents’, interns’, and medical students’ attitudes towards emigration [2]. The study found that 75% of the 519 participants would emigrate if the opportunity presented itself [2]. Truthfully, this attitude is completely justified. Given the significant instability of life in Lebanon, emigration provides financial, emotional and psychological benefits that are difficult to overlook. This goes without mentioning the post-traumatic stress that many physicians associate with Beirut, particularly after the August 4^th^ Beirut Port Explosion [2].

On the other hand, however, Lebanon has witnessed significant developments in pediatric cardiology over the past decade [5]. After so much progress, it would be a shame to allow the continuation of our regression, especially given our moral obligation to treat these children despite their socioeconomic standing. Despite the catastrophic outcome of this phenomena, a silver lining exists. Through examining Lebanon’s response to catastrophe, many lessons were learnt regarding the protocols that assisted or fell short in crises management. Similarly, reflection enabled the identification of protocols that could have aided a more effective response had they existed. Thus, many studies now exist examining the protocols that could best equip global hospitals with the tools needed to protect themselves from sudden, massive crises. This includes the prevention of severe workforce shortages in the face of economic destruction [6].

Though philanthropic organizations exist both locally and internationally to provide aid to patients from impoverished areas, there still remains an overwhelming majority that are left to their own devices. Unfortunately, with so much going wrong in the country, this issue falls low on the government’s list of priorities. Nevertheless, Lebanon is in need for well-planned financial strategies combining philanthropic organizations, private university hospitals, and the public sector under a collective movement to allow pediatric cardiac programs to function without financial loss. In addition to implementing a strategic plan to improve the management of newborns with CHD, it is also important to apply preventive measures in order to decrease their incidence. This will be possible by:

- Improving access to prenatal care to identify potential risk factors for CHD and ensure early diagnosis and treatment.
- Addressing risk factors such as maternal smoking, alcohol consumption, and certain infections during pregnancy.
- Immunization against certain infections such as rubella.
- Improving the maternal nutritional status during pregnancy.
- Promoting healthy lifestyles, such as regular exercise and a healthy diet.
- Increasing awareness of the risk factors and symptoms of CHD.

## Data Availability

Not applicable.

## Abbreviations

USA: United States of America
NGO: Non-governmental organizations
